# Screening and brief intervention for drug use in primary care: the Assessing Screening Plus brief Intervention’s Resulting Efficacy to stop drug use (ASPIRE) randomized trial

**DOI:** 10.1186/1940-0640-8-S1-A61

**Published:** 2013-09-04

**Authors:** Richard Saitz, Tibor P Palfai, Debbie M Cheng, Daniel P Alford, Judith A Bernstein, Christine A Lloyd-Travaglini, Seville M Meli, Christine E Chaisson, Jeffrey H Samet

**Affiliations:** 1 Clinical Addiction Research and Education (CARE) Unit, Section of General Internal Medicine, Boston University School of Medicine and Boston Medical Center, Boston, Massachusetts, USA; 2 Department of Epidemiology, Boston University School of Public Health, Boston, Massachusetts, USA; 3 Department of Psychology, Boston University College of Arts and Sciences, Boston, Massachusetts, USA; 4 Department of Biostatistics, Boston University School of Public Health, Boston, Massachusetts, USA; 5 Department of Community Health Sciences, Boston University School of Public Health, Boston, Massachusetts, USA; 6 Data Coordinating Center, Boston University School of Public Health, Boston, Massachusetts, USA

## Background

The efficacy of universal screening and brief intervention (SBI) for drug use among primary care (PC) patients is unknown.

## Methods

In this randomized trial (the Assessing Screening Plus brief Intervention’s Resulting Efficacy to stop drug use (ASPIRE) study) we tested the efficacy of a brief negotiated interview (BNI), and an adaptation of motivational interviewing (AMI), compared to no BI. Primary care patient participants had Alcohol, Smoking and Substance Involvement Screening Test (ASSIST) drug specific scores of ≥4. Primary outcome at 6 months was number of days use of the drug of most concern (DOMC) in the past 30 days.

## Results

Of 528 subjects, DOMC was: marijuana 63%, opioid 17% (prescription opioid 11%), and cocaine 19%. ASSIST score was ≥27 (consistent with dependence) for 18%, mean days DOMC use (of 30 days) was 14.4. At 6 months, 98% completed follow-up and mean days DOMC use was 14.0. Mean adjusted days use of the DOMC at 6 months (negative binomial regression) was 11.5 (no BI) vs. 11.2 (BNI) (incidence rate ratio (IRR) 0.97, 95% CI 0.77-1.22) and 12.1 (AMI) (IRR 1.05, 95% CI 0.84-1.32) (p=0.81 for both comparisons vs. no BI). There were also no significant effects in analyses stratified by DOMC or ASSIST score.

## Conclusions

In this trial, BNI and AMI did not have efficacy for decreasing drug use. If other trials yield consistent results, widespread implementation of drug screening and BI should be reconsidered, and research should focus on alternative ways to address drug use and consequences in primary care settings. Funding was provided by the US National Institutes of Health (National Institute on Drug Abuse and National Center for Advancing Translational Sciences).

